# CasPDB: an integrated and annotated database for Cas proteins from bacteria and archaea

**DOI:** 10.1093/database/baz093

**Published:** 2019-08-14

**Authors:** Zhongjie Tang, ShaoQi Chen, Ang Chen, Bifang He, Yuwei Zhou, Guoshi Chai, FengBiao Guo, Jian Huang

**Affiliations:** 1 Center for Informational Biology, University of Electronic Science and Technology of China, Chengdu 611731, China; 2 School of Medicine, Guizhou University, Guiyang 550025, China

## Abstract

Clustered regularly interspaced short palindromic repeats (CRISPR) and associated proteins (Cas) constitute CRISPR–Cas systems, which are antiphage immune systems present in numerous bacterial and most archaeal species. In recent years, CRISPR–Cas systems have been developed into reliable and powerful genome editing tools. Nevertheless, finding similar or better tools from bacteria or archaea remains crucial. This requires the exploration of different CRISPR systems, identification and characterization new Cas proteins. Archives tailored for Cas proteins are urgently needed and necessitate the prediction and grouping of Cas proteins into an information center with all available experimental evidence. Here, we constructed Cas Protein Data Bank (CasPDB), an integrated and annotated online database for Cas proteins from bacteria and archaea. The CasPDB database contains 287 reviewed Cas proteins, 257 745 putative Cas proteins and 3593 Cas operons from 32 023 bacteria species and 1802 archaea species. The database can be freely browsed and searched. The CasPDB web interface also represents all the 3593 putative Cas operons and its components. Among these operons, 328 are members of the type II CRISPR–Cas system.

## Introduction

Clustered regularly interspaced short palindromic repeats (CRISPR) and their associated proteins (Cas) constitute CRISPR–Cas systems (1). Since the first biological evidence for the participation of CRISPR–Cas systems in adaptive immunity was reported in 2007 (2, 3), these systems have gradually become a research hotspot (4, 5), further expanding biotechnological toolkits, and especially revolutionizing the genome editing technology (6). As the adaptive immunity system of archaea and bacteria, CRISPR–Cas systems imprint exogenic elements as memories by inserting them into an array of CRISPR repeats, and then the inserted fragment can be used in the form of guide CRISPR RNAs (crRNAs) that cooperate with Cas proteins to recognize and shear the cognate viral genome or plasmid upon new infection (7). For example, the CRISPR–Cas9 technology derived from the *Streptococcus pyogenes* engineers the dual tracrRNA:crRNA into a single guide RNA (sgRNA) that retains two critical features: a 20-nucleotide sequence at the 5′ end of the sgRNA that undergoes Watson–Crick base pairing with any DNA sequence of interest and a double-stranded structure at the 3′ side of the guide sequence that binds to Cas9 (8, 9). Therefore, the exploration of the mechanisms of CRISPR–Cas systems requires understanding and characterization of new Cas proteins, and this may also be a rational start point for the discovery of novel scissors for genome-editing technology.

A wealth of structural and functional information on core Cas proteins has accumulated through the continuous implementation of large-scale research (10–13) and has facilitated the classification of CRISPR–Cas systems. At present, class 1 CRISPR–Cas systems include the CRISPR–Cas type I, type III and type IV systems. Their effector modules are composed of several Cas proteins (10, 14). The class 2 CRISPR–Cas systems mainly include CRISPR–Cas type II, type V and type VI systems. Their effector module consists of a single, multidomain Cas protein. The common class 2 type effector includes Cas9 (Csn1, II), Cas12a (Cpf1, VA), Cas12b (C2c1, VB), Cas13a (C2c2, VI-A), Cas13b (C2c6, VI-B) and Cas13c (C2c7, VI-C). As for Cas9, it contains two unrelated nuclease domains, RuvC and HNH, responsible for cleavage of the displaced (non-target) and target DNA strands, respectively (10, 14–16). Totally, the annotation and recognition of Cas proteins in bacteria and archaea are necessary not only for correctly inferring the type of CRISPR–Cas systems but also for supporting the development of novel scissors for gene-editing technology.

Unfortunately, the UniProt protein resource contains only 287 bacterial and archaeal Cas proteins that have been manually reviewed (17). Many novel Cas proteins await discovery. Thus, predicting Cas proteins from bacteria and archaea proteomic data and integrating them into a specific database is highly important. Some of the web services and databases about the CRISPR–Cas system have been developed. For example, the well-known CRISPRFinder (18) is a web tool for searching CRISPRs, and CRISPRCasFinder (19) supports to search CRISPRs and the Cas gene. Relevant databases include CRISPRdb (20), a data resource of CRISPRs, and CrisprGE (21), a useful web resource for genome editing that provides comprehensive information of the genome editing approach, including target sequence, modification length and CrisprID. At present, no specific database is available for annotating and housing Cas proteins. Therefore, a comprehensive data resource for potential Cas proteins and Cas operons from bacteria and archaea is still necessary. Some computational methods have been used for the rapid annotation of protein sequences. The Hidden Markov Model (HMM) technique (22) is one of the methods used to identify potential Cas proteins from proteomic data. We built the HMMCAS website (http://i.uestc.edu.cn/hmmcas/index.html) to predict Cas proteins (23). Herein, we identified proteome-wide putative Cas proteins across bacteria and archaea using the HMM technique, screened Cas protein clusters and the neighboring CRISPR array through CRISPRFinder and inferred the potential CRISPR–Cas system type. All the information was stored in a database called CasPDB, which is short for Cas Protein Data Bank. It can be conveniently and flexibly browsed, searched and downloaded. The putative Cas proteins and operons can be easily visualized.

## Materials and Methods

**Figure 1:**
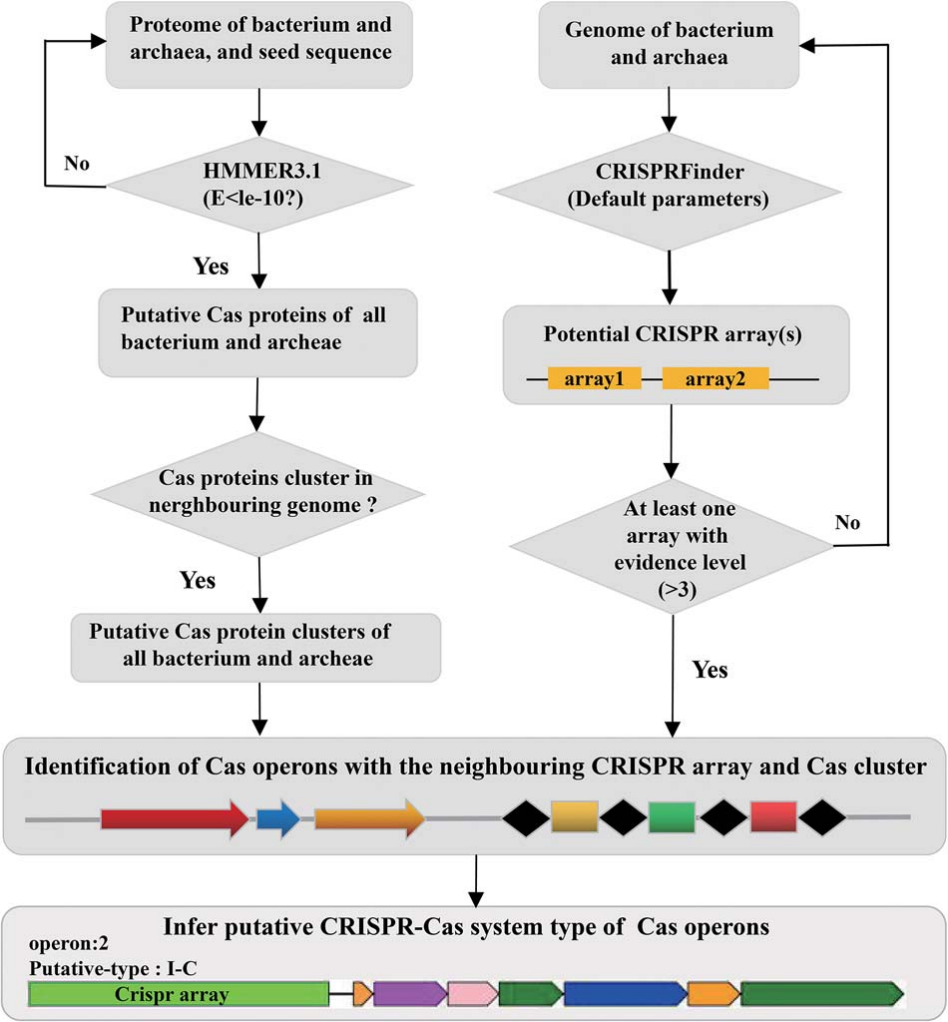
The construction of CasPDB.

The construction of CasPDB is illustrated in Figure 1.

### Data Collection and Model Construction

Firstly, we downloaded 101 and 38 CRISPR-associated multiple sequence alignments or seed alignments of protein families from the TIGRFAMs (version 15.0) (24) and Pfam (version 30.0) databases (25), respectively. We also retrieved 24 novel Cas seed alignments collected from http://omics.informatics.indiana.edu/mg/CAS (26). Secondly, we used these CRISPR-associated seed alignments to construct the HMMs by the hmmbuild program with default parameters. We then employed the HMMs to predict all candidate Cas proteins from the whole proteome of different bacterial and archaeal species collected from GenBank (all data for archaea is downloaded from https://ftp.ncbi.nih.gov/genomes/genbank/archaea/, and that of bacteria is from https://ftp.ncbi.nih.gov/genomes/genbank/bacteria). The hmmbuild program (using the gathering cutoff) in HMMER3.1 (22,27) is implemented by Python scripts (https://www.python.org/).

### Identification of Putative Cas Proteins and Cas Operons

Using the hmmscan homology search program to compare the HMM modules (HMMs) against the complete proteomic sequence data of 32 023 and 18 021 bacterial and archaeal species, we obtained 257 745 putative Cas proteins. If two or more Cas proteins in a proteome are not spaced by three or more non-Cas proteins, these Cas proteins form a Cas protein cluster. We got a total of 30 495 Cas protein clusters. We also collected 287 Cas proteins from UniProt which are manually reviewed (17). The genome data of bacteria and archaea is from the same URL of proteome data. To further identify potential Cas operons in bacteria and archaea, we used CRISPRFinder (18) to genome-wide screen potential CRISPR arrays on the genome adjacent to the Cas protein clusters. If a CRISPR array locates to a Cas protein cluster within 5000 bp, we define the Cas protein cluster and the neighboring CRISPR array a Cas operon. Finally, based on the composition of the Cas proteins in the cluster and their relative genomic location, the types of possible CRISPR–Cas systems can be judged. For example, the cluster with Cas9–Cas1–Cas2–Csn2 in the Cas operon will be defined as the type II-A system, clusters with Cas3 belong to type I and Cas9 type II, Cas10 type III, etc. All putative Cas proteins were mapped back to the genome, and the corresponding protein accession number was provided (Figure 1).

### Nomenclature Standardization and Classification

We standardized the species name and taxonomy ID in accordance with the NCBI Taxonomy (https://www.ncbi.nlm.nih.gov/Taxonomy/). Protein names and corresponding accession numbers were also standardized with NCBI GenBank. The putative CRISPR–Cas system type was identified on the basis of the core putative Cas proteins and their relatively genomic location in the Cas operon.

### Maintenance and Quality Assurance

As described above, we developed a pipeline to identify putative Cas proteins and Cas operons according to the genome and proteome data stored at NCBI. The Cas proteins were determined using the HMMER program based on the Cas seed files collected from multiple resources. The species name, taxonomy ID, protein names and relevant accession numbers were in accordance with the corresponding databases of NCBI. The putative type of Cas operons was named according to the newest nomenclature of CRISPR–Cas systems. As our pipeline strictly agrees with the data standards of NCBI, the data quality of CasPDB can be assured. We plan to run the pipeline and update the CasPDB database annually to keep up with the update of the genome and proteome data of archaea and bacteria, the new collections of the Cas seed files, the new version of relevant NCBI databases and the new rules for the classification of CRISPR–Cas systems. The online version of the CasPDB database is always the newest release. All previous releases will be archived and can be downloaded from the download page.

### Implementation of Web Interface

All metadata in CasPDB were stored in a MySQL database. The CasPDB website is implemented with the HyperText Markup Language (HTML, https://www.w3.org/), Cascading Style Sheets (CSS), and JavaScript. ECharts 3 (http://echarts.baidu.com/) were used for the web-based visualization of Cas operons.

## Results

### Data Statistics

**Figure 2:**
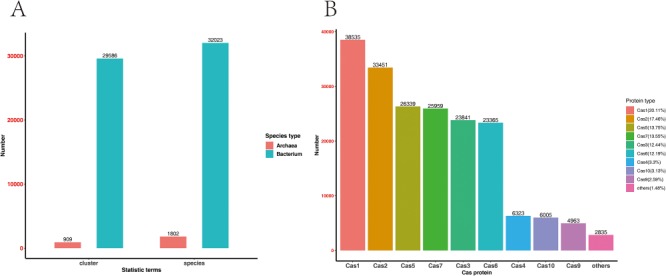
Statistics of CasPDB. The number of bacterial and archaeal species and their putative Cas operons **(A)**. Statistical distribution of each type of Cas proteins **(B)**.

At present, the CasPDB database contains 287 reviewed Cas proteins, 257 745 putative Cas proteins, 30 495 Cas protein clusters and 3593 Cas operons from 32 023 bacterial species and 1802 archaeal species (Figure 2A). In all candidate Cas proteins, Cas1 accounts for 20.11%, Cas2 accounts for 17.46% and Cas3, Cas5 and Cas6 accounts for more than 10%. One of the major editing proteins, namely Cas9, only accounts for 2.59% of the candidate proteins (Figure 2B). No putative Cas12 or Cas13 proteins were identified because these types of Cas proteins lack seed alignments at the time when we ran the pipeline. Moreover, we identified 328 putative operons of the type II system. These CRISPR–Cas9 systems can be used for further analysis on genome engineering.

### Guidance of CasPDB

**Figure 3:**
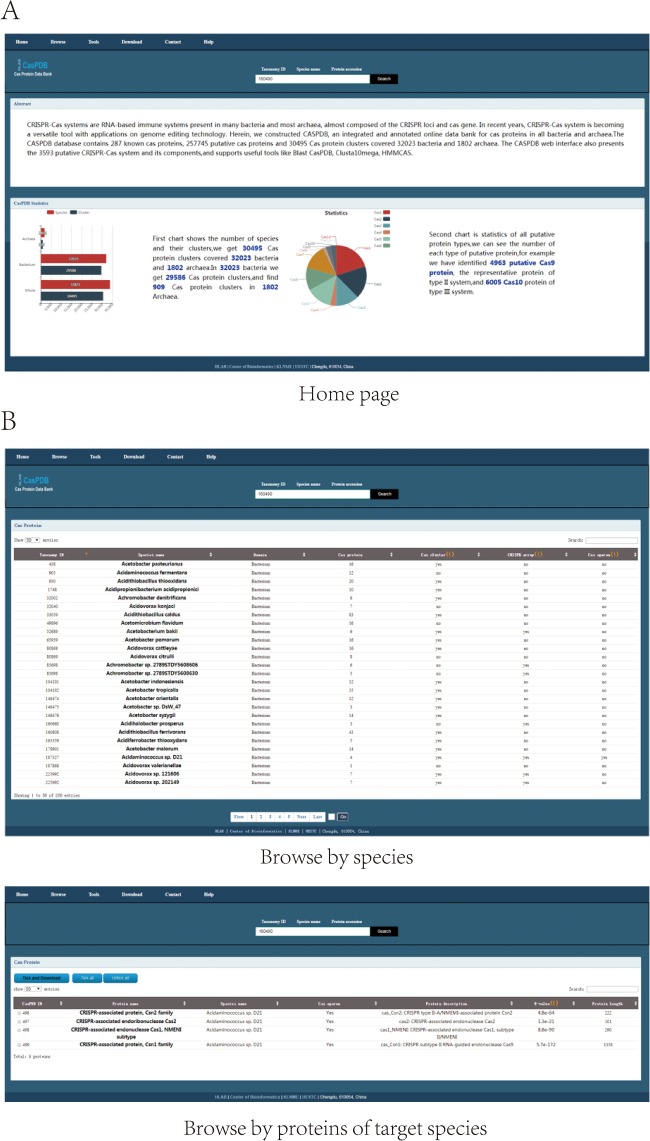
Home and browse pages. Search option and home page **(A)**. Browse page with all Cas proteins. The bottom section of the page shows protein distribution in bacteria **(B)**.

**Figure 4:**
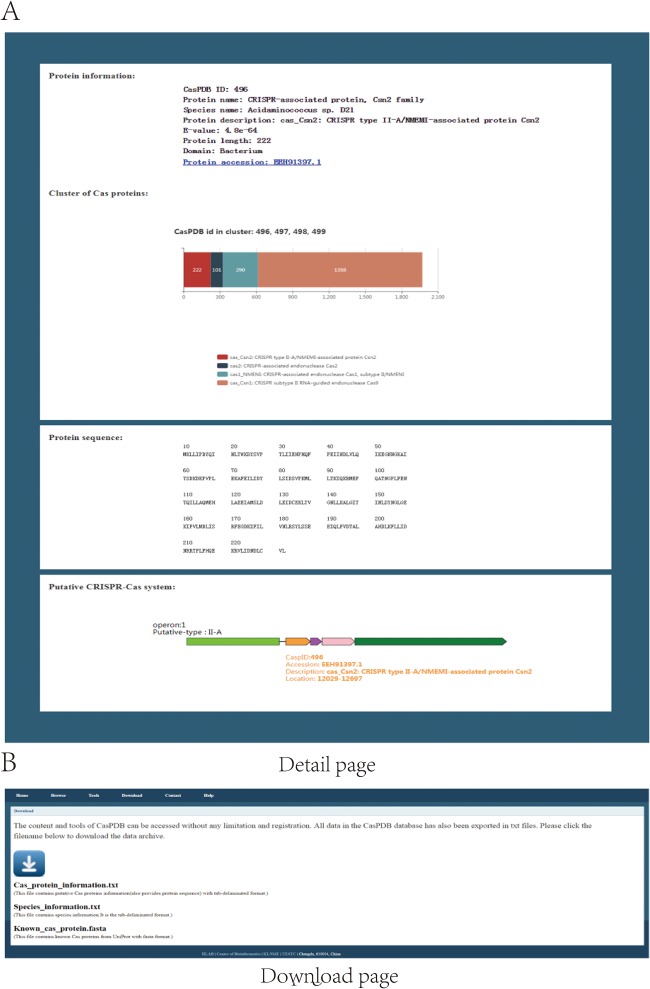
Detail and download pages. The detail page shows basic information of protein and operons **(A)**. Download option for all Cas proteins and proteins of selected items **(B)**.

The CasPDB web interface enables the searching, browsing and downloading of putative Cas proteins and operons from bacterial and archaeal species. Firstly, the search panel accepts three types of key words: Taxonomy ID, Species name and Protein accession. Users can input the taxonomy ID or scientific species name of the NCBI Taxonomy database, or protein accession of NCBI GenBank to find Cas proteins. The home page describes the basic information of CasPDB and shows statistics of the Cas proteins, Cas protein clusters and species (see in Figure 3A). As shown on the browse page, all putative Cas protein numbers and putative CRISPR–Cas system distributions across all bacteria and archaea have been listed with the following information: Taxonomy ID, Species name (scientific name of bacteria or archaea), Domain (Cas proteins from Bacteria or Archaea), Cas protein (number of putative Cas proteins in target species), Cas cluster (includes putative Cas protein clusters or not), CRISPR array (includes putative CRISPR array or not) and Cas operon (includes Cas operon or not). Upon further clicking the species name in the browse page, all putative Cas proteins of target species will be listed with the following information: CasPDB ID (CasPDB ID of putative Cas protein), protein name (putative Cas protein name), species name, Cas operon, protein description (detailed annotation information of putative Cas protein), E-value (the statistical significance of the possibility that a candidate protein is a Cas protein) and protein length (see Figure 3B). Secondly, the detail page of putative Cas protein will display protein information, cluster of Cas proteins if any, protein sequence and putative CRISPR–Cas system, if any. Protein information includes the basic protein information and protein accession number link to the NCBI GenBank database. A cluster of Cas proteins figures genomic neighboring Cas proteins, labeled with different colors and detail protein information. The putative CRISPR–Cas system displays the element of the Cas operon, includes the Cas protein cluster and CRISPR array and is labeled with corresponding putative Cas protein basic information and hyperlinked to the corresponding CasPDB page (see Figure 4A). Finally, on the download page, users can download all putative Cas proteins, species information and known Cas proteins from UniProt across all bacteria and archaea with their basic information in tab-delimited format or FASTA format. Besides, users can also download only the items of interest through checking the box in front of the CasPDB ID when browsing a species or on the search result page (Figure 4B). The CasPDB database also provides additional information and an external interface of other prediction tools for putative Cas proteins, such as Clustal Omega, Blast CasPDB and HMMCAS (23). The tool Blast CasPDB can blast the user’s query protein sequences against the CasPDB database. By comprehensively using these tools, users can conveniently obtain more complete characteristics of Cas proteins.

### Identification and Presentation of Putative Cas Operons

In CasPDB, we identified 3593 Cas operons, among which 328 belongs to the type II CRISPR–Cas systems. In the detail page, the picture of the putative CRISPR–Cas system shows operon (operon ordinal in target species), operon-putative type (putative CRISPR–Cas system type of Cas operon), Cas proteins (different color arrows) and CRISPR array (green rectangle). When the mouse moves over any Cas protein icon, the detail information including CasPDB ID, Accession, Description, Location (genomic location of putative Cas protein) will be represented. Besides, clicking the protein icon in the operon figure, the page will skip to the detail page of the corresponding protein in CasPDB. When the mouse moves over the CRISPR array, the detail information with Element (type of element in operon), Spacer_num (number of spacer), Description (repeat sequence in CRISPR), Location (genomic location of CRISPR array) will be displayed (see in Figure 4A).

## Discussion

Over the past 10 years, with the deep understanding of the CRISPR–Cas systems, RNA-guided DNA endonucleases, such as Cas9 and c2c2, have been programmed to be genome editing tools (9, 28). The continuous expansion of CRISPR–Cas systems has provided prospective novel genome-editing tools (29). Unfortunately, the numbers of experimentally validated and annotated Cas proteins remain insufficient for meeting the need of researchers, and no specialized database for Cas proteins has been designed. Hence, putative Cas proteins from the whole proteome of bacterial and archaeal species should be studied systematically. In our work, we identified putative Cas proteins from proteomic data based on the Cas-related seed alignments of experimentally characterized Cas proteins, and annotation information of basic protein information, amino acid sequence and genomic location for each putative Cas protein. We further identified the genome-wide CRISPR array and screened their genomic neighboring (<5000 bp) Cas protein clusters, and formed putative Cas operons. According to the core Cas proteins and their relative order in Cas operons, we classified the potential CRISPR–Cas system type of Cas operons. The CasPDB database will help users to find the potential CRISPR–Cas systems in bacteria and archaea and further infer other elements in the CRISPR–Cas systems, such as tracrRNA and PAM. The comprehensive analysis of these elements in the same CRISPR–Cas system and the optimization of their annotated information may be a highly effective approach for identifying new scissors for genome editing (30, 31). We believe that CasPDB is a valuable data resource for bacterial and archaeal Cas proteins and can aid in the discovery of new scissors for genome editing.

In conclusion, we provided a user-friendly website with data retrieval capabilities and browsing, searching and downloading options to facilitate data access. In the future, we will continue to collect new CRISPR-associated multiple sequence alignments and seed alignments and integrate proteome and genome data of new bacteria and archaea species to screen new potential Cas proteins, further combining the genomic and proteomic data to identity the putative Cas proteins and other elements of CRISPR–Cas systems, to provide a complete perspective for understanding what role CRISPR–Cas systems play and to support the use of valuable CRISPR–Cas systems or Cas proteins as new scissors for genome-editing technology.

## Notes

**Database URL**: http://immunet.cn/caspdb/
